# Scenario evolution modeling and probabilistic assessment of seawater intrusion accident in ports: An integrated framework combining disaster theory and multi-method simulation

**DOI:** 10.1371/journal.pone.0334696

**Published:** 2025-10-31

**Authors:** Zhen Qiao, Xiaotiao Zhan

**Affiliations:** Safety Quality Technology Research Center, China Waterborne Transport Research Institute, Beijing, China; Malankara Catholic College, INDIA

## Abstract

As a typical marine disaster, seawater intrusion accidents have posed a serious threat to port production safety due to the double rise in the occurrence frequency and damage intensity. In favor of effectively controlling the scope of disaster impact and formulating more targeted emergency plans, it is particularly significant to carry out accident scenario evolution analysis. Based on the disaster system theory, this study constructed a model for the evolution of seawater intrusion accident scenarios in ports and clarified the probability of occurrence concerning each accident scenario by utilizing qualitative and quantitative methods. The main conclusions of this study were as follows: According to the theoretical framework of “disaster-causing body, disaster-affected body, and disaster-resistant body”, typical scenarios, such as concrete structure erosion and power supply interruption, were identified by scenario element method. By coupling the Petri net, cloud model, and Monte Carlo model, the quantitative derivation of evolutionary paths was realized, which not only retained the organic link between qualitative cognition and quantitative expression but also guaranteed the reliability of the results through ten thousand iterations. The probability grading system of accident scenarios was formed by combining the quantitative results. Among them, S11(Large equipment such as gantry and shore bridges stopped working due to power supply interruption) had the highest probability, with the corresponding value of 57.2%, and was in the “Moderately Likely” level according to the preset interval level. The research can provide a scientific basis for port enterprises to optimize the preparation with regard to emergency plans and improve the post-disaster recovery strategy, helping advance the comprehensive disaster prevention and mitigation capacity of ports.

## 1 Introduction

Under the influence of multiple factors, including dramatic changes in the global climate system, the intensification of human activities, and continuous sea level rise, seawater intrusion—a typical marine disaster—has shown a significant increase in frequency and damage intensity [1–5]. For port enterprises, the danger of such disasters is not only the salinization of groundwater and undermines the infrastructure [6–8], but also the paralysis of the production system, which directly threatens the safety of enterprise operations [9,10]. In October 2024, for instance, in China’s Yellow Bohai Sea, sudden seawater intrusion caused flooding of yards, submergence of equipment, and other disasters at important hubs such as Yingkou port and Panjin port. Therefore, for the sake of systematically improving disaster prevention and mitigation capabilities, it is necessary to construct a scenario evolution model for seawater intrusion accidents in ports.

In fact, scenario evolution analysis has been widely utilized in various industries, such as financial risk management [11,12], fire and explosion accidents [13–18], natural disaster analysis [19–22], and coal mine safety management [23–25]. In the field of engineering safety, Li et al. [26] conducted dynamic risk identification for shield tunnel construction safety by coupling scenario evolution analysis, data-driven technology, and knowledge mapping methods. For university laboratory safety, Liu et al. [16] constructed a coupled Bayesian network-case inference model to reveal the logical relationship between fire and explosion scenarios, and realized the explicit expression of risk conduction paths. In terms of safety governance, Zhao et al. [24] built a scenario-matching algorithm for coal mine outburst accidents by analyzing 468 historical cases, which was applied to improve the response efficiency of outburst accidents. Ma et al. [20] focused on secondary disasters of earthquakes, and introduced the scenario evolution analysis into the Natech accident to compose a quantitative regional risk assessment model. At the level of methodological innovation, Yao et al. [15] proposed a risk coupling analysis framework to reveal the interaction mechanism of fire risk elements in urban communities by dynamically optimizing the scenario evolution process. Song et al. [27] incorporated the scenario evolution theory with the Petri net and Fuzzy theory for identifying key evolutionary nodes and triggering behaviors concerning the 2020 coronavirus outbreak. Additionally, Wang et al. [28] extended scenario evolution analysis to broader application scenarios, i.e., to realize innovative product development by constructing scenario evolution chains.

Switching the perspective to the research concerning seawater intrusion. Currently, most scholars mainly focus on the construction of a warning system for monitoring seawater intrusion [2,3,29,30]. Special attention has been paid to the pollution of groundwater systems and the risk of salinization by seawater intrusion [31–34]. Fu et al. [31] combined hydrochemical characterization and isotope tracer techniques to carry out a multidimensional impact assessment on the coastal areas of China to reveal the impact mechanism of seawater intrusion on the groundwater system. Xiong et al. [32] established a prediction model for assessing the risk of seawater intrusion based on multi-source environmental parameters and data mining techniques. Wang et al. [33] examined the impact of seawater intrusion on coastal cities from the rainfall infiltration perspective, and quantified the optimal rainfall infiltration volume for mitigating seawater intrusion intensity. Ez-zaouy et al. [35] assessed the vulnerability of groundwater systems to seawater intrusion by integrating nine key parameters and classified this vulnerability into five grades for clear characterization. Li et al. [36] developed a seawater intrusion risk assessment model via coupling the Fuzzy Delphi Analytic Hierarchy Process (FDAHP) with the entropy weight method, incorporating 14 critical influencing factors. In addition, Zhang et al. [37] focused on seawater intrusion-induced hydrogeological changes and explored the correlations between total soil salt content, groundwater chloride concentration, and groundwater depth.

Through the combing and summarizing of existing literature, it is found that academics have accumulated certain research results in the field of seawater intrusion and scenario evolution analysis. However, there were two deficiencies in the existing study: first, the organic integration of seawater intrusion and accident scenario evolution modeling weren’t carried out; and, second, there was a lack of logical framework for investigating seawater intrusion as a typical marine disaster from the perspective of ports. In view of this, based on the operational features of ports, the paper built an accident scenario evolution model for seawater intrusion, and applied the Petri net, cloud mode, and Monte Carlo simulation to quantitatively assess the probability of various types of disaster scenarios. The research aims to provide theoretical support for port enterprises to formulate targeted emergency response plans and optimize post-disaster resumption decision-making, which is helpful for upgrading of port’s production safety management system.

## 2 Scenario evolutionary paths for seawater intrusion accident in ports

In the context of responding to major mass emergency incidents, a scenario referred to the sudden high-impact crisis encountered by decision-makers and its dynamic evolution process. This scenario wasn’t static; instead, driven by multiple variables, it gradually transitioned from an initial state to subsequent new scenarios under the interaction of natural laws and human intervention—a process defined in academia as scenario evolution.

The components of a disaster scenario could be broken down into the following three parts: the disaster-causing body, the disaster-affected body, and the disaster-resistance body. The disaster-causing body was a natural entity with disaster-causing potential. The disaster-affected body referred to the object affected by the disaster-causing body, including many aspects of human society and natural ecology. The disaster-resistant body referred to the primary body of disaster response, incorporating professional rescue teams, emergency decision-making institutions, etc.

**Fig 1:**
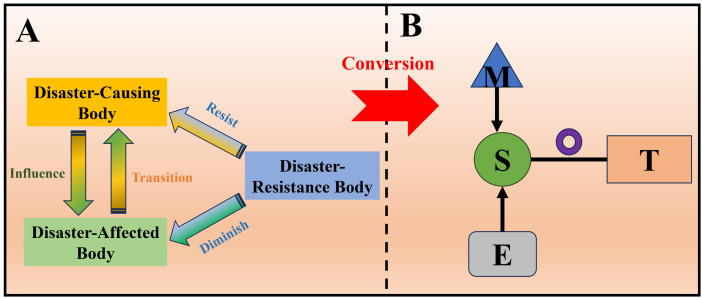
Basic theories and methodologies in scenario evolution paths: A-“disaster-causing body, disaster-affected body, and disaster-resistant body” theory; B-scenario element representation method.

The response characteristics among the three was displayed in Fig 1A. The disaster-causing body was the source of the disaster, and its activities would have an impact on the disaster-affected body. In turn, damage to the disaster-affected body may be transformed into the disaster-causing body, triggering secondary accidents. The disaster-resistant body, on the other hand, ran through the entire disaster process, from disaster monitoring and warning, emergency response to post-disaster recovery and reconstruction.

To promote the transformation of situational evolution theory into practice and facilitate the subsequent qualitative and quantitative analysis, this paper utilized the visualization method of situational elements. The method was based on the system of “disaster-causing body, disaster-affected body, and disaster-resistant body”, and investigated unconventional sudden-onset disaster events from the perspective of emergency management, as presented in Fig 1B.

Specifically, the method broken down the disaster evolution process into five pivotal elements: first, the situation state (S), i.e., the initial situation at the time of the disaster; second, the emergency response goal (T), i.e., the goal to be achieved in response to the disaster; third, the measure (M), i.e., specific actions taken to achieve the emergency response goal; fourth, the external environment (E), i.e., the environmental factors surrounding the disaster at the time of its occurrence; and fifth, the evolution of the disaster itself (◎), i.e., the process of development and change of the disaster itself.

**Fig 2:**
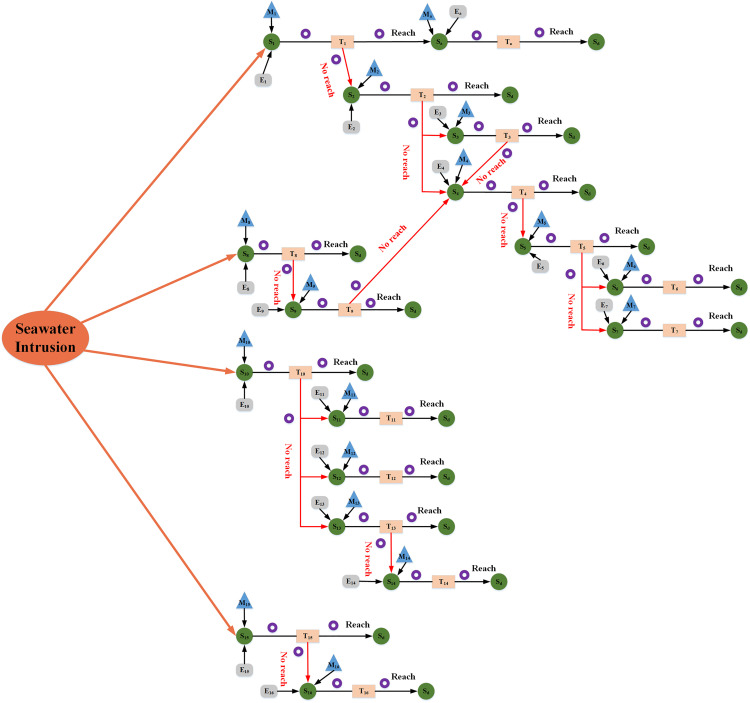
Scenario evolution paths for seawater intrusion accident in ports according to the scenario element method.

According to the scenario element representation, the scenario evolution path after a seawater intrusion accident in the port was plotted in Fig 2. The evolutionary directions of scenarios were divided into two categories, namely, favorable and unfavorable, and usually didn’t remain constant. The determination of the evolutionary direction depended on whether the evolution met the expectations of the decision-maker. If it met the expectations, it was considered to evolve in a favorable direction; conversely, if it didn’t meet the expectations, it was considered to evolve in an unfavorable direction.

Fig 2 and Table 1 illustrated the evolution of the scenario after a seawater intrusion accident in the port. At the initial stage, the seawater intrusion would directly erode the concrete structure of the quay. If scientific and effective emergency response measures were taken, the situation would enter a controlled steady state (denoted as Sa), after which the effects of seawater intrusion gradually subsided and the structural damage wouldn’t continue to expand.

**Table 1:** Key scenarios and their implications in port seawater intrusion accidents.

Scenario	Scenario	Scenario
Seawater erosion of concrete structure of wharf S1	Uneven foundation settlement S2	Serious waterlogging problem S3
Damage to the wharf’s foundation structure S4	Displacement and tilting of large-scale loading and unloading equipment, such as gantry and quay bridge S5	Large-scale loading and unloading equipment tipping over to the sea surface S6
Large-scale loading and unloading equipment tipping over to the land surface S7	Displacement or breakage of fenders S8	Damage to structure due to impacts by staying ships on the wharf S9
Damage to substations causing disruption of power supply S10	Large equipment such as gantry and shore bridges stopped working due to power supply interruption S11	Failure of shore power system affecting normal operation S12
Communication equipment such as base stations and fiber optics damaged due to power outage S13	Inability to update the status of automation equipment in real time S14	Damage to high-pole lamps S15
high-pole lamps shifted to the land surface resulting in casualties and loss of cargo S16		

However, if the initial disposal measures were flawed, the situation would progress in an unfavorable direction to scenario S2, i.e., uneven settlement of the foundation. At this stage, if no remedial measures were taken, the situation may continue to deteriorate along two paths: firstly, the waterlogging problem was serious (S3), with the depth of waterlogging continuing to increase in the operation area and the drainage system being overloaded; second, the terminal infrastructure was damage (S4), which was manifested by cracking of concrete elements and reduction in the bearing capacity of the piled. It was worth noting that there was a one-way correlation between S3 and S4, namely, severe waterlogging exacerbated structural damage.

When the scenario evolved to the S4 stage, the continuous deterioration of the foundation conditions would directly threaten the safety of the port’s core loading and unloading equipment. Under the dual action of structural instability and water erosion, large equipment such as gantry machines and shore bridges may be displaced or tilted (S5). If the equipment reinforcement and structural repair measures were still not implemented at this stage, it would eventually lead to two extreme consequences: one was the complete failure of the support system, and the equipment would be titled to the sea surface (S6); the other was the uneven settlement of the foundation, and the equipment would be tilted to the land side (S7).

Seawater intrusion was prone to induce displacement or fracture of pier fenders (S8). When the emergency response was insufficient, and the tide level continued to be abnormal, the moored vessel would strike the pier due to fender failure, causing structural damage to the pier (S9). As the hydrodynamic effect intensifies, the above damage scenario would transform to S4.

In addition, seawater intrusion would also attack the power supply and distribution system of port terminals, resulting in damage to power substations, such as transformer flooding and insulation failure of high-voltage switchgear, which would lead to power supply interruption (S10). If the emergency plan wasn’t activated in time, the following three secondary risks would triggered: first, large-scale equipment such as gantry machines and shore bridges would stop working due to power supply interruption (S11); second, the shore power system would malfunction, which would affect the normal operation (S12); and third, the base station, fiber optic, and other communication equipment would be damaged due to the power outage (S13). In particular, it should be noted that once the duration of communication interruption reached a critical value, it would provoke the broken chain of automated equipment status monitoring, which in turn would lead to the disorganization of operation procedures (S14). This type of systemic risk could be far more disruptive to the port’s production order than a single equipment failure.

Lighting high-pole lamp base immersed in seawater was easy to cause its corrosion. If the inclination angle of the pole body exceeded the safety threshold, there was a potential danger of the high mast lamp falling toward the operation area and the yard (S15). Such failing accidents not only directly jeopardized the lives of the personnel on site, but also caused damage to cargo and transportation vehicles as a result of the movement of water currents (S16).

## 3 Quantitative assessment methods for scenario evolutionary pathways

### 3.1 Petri net construction

The scenario evolution path of port seawater intrusion accidents, constructed based on the scenario element method, already had a visual presentation capability. However, it remained at the qualitative description level, lacking quantitative analysis methods to deepen the analysis. In view of this, this study introduced Petri net theory and converted the existing scenario evolution diagram into a Petri net model, which served as a carrier for transforming qualitative analysis into quantitative analysis.

**Fig 3:**
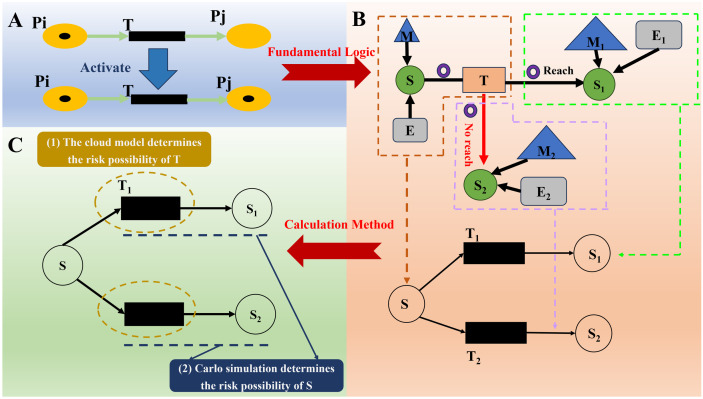
Petri nets in the quantitative analysis of scenario evolution paths: A-underlying logic; B-transformation paths; C-computational methods.

The structure of a Petri net was essentially a directed bipartite graph, consisting of two basic elements, the place (represented by a circle symbol) and the transition (represented by a rectangle symbol). These elements were related through directed arcs, which characterized the input and output relationships between the elements. Concerning a better fit of chained features of scenario evolution, an extension of the classical Petri net was needed. In other words, the place represented the individual state nodes, the transition represented the triggering mechanisms of events, and the Token represented the occurrence of events. That is to say, when a Token appeared in a certain place, it indicated that the state represented by that node had been reached, as illustrated in Fig 3A.

Fig 3B illuminated the realization path of the scenario evolution figure to Petri net in detail. First, the core elements of scenario evolution, i.e., scenario state, emergency response objective, disposal plan, external environment, and natural evolution of accidents, were uniformly mapped into the numbered places in Petri nets. On this basis, the two basic forms of accident evolution were materialized as two evolutionary branches of the place. Specifically, the evolution of place S to S1 via transition T_1_ corresponded to the positive turn of controlled risk, while the extension of place S to S2 via transition T_2_ portrayed the negative evolution of the continuous spread of the crisis.

Fig 3C highlighted the quantitative analysis method based on Petri net. Its technical path could be decomposed into two progressive stages. In the first stage, the cloud modeling theory was utilized to quantitatively assess the probability of occurrence of all transitions, including the establishment of the range of values and activation thresholds for the transition. After entering the second stage, the Monte Carlo simulation was imported to assess the probability of the dynamic process of state transfer to a new place after the triggering of a change.

**Fig 4:**
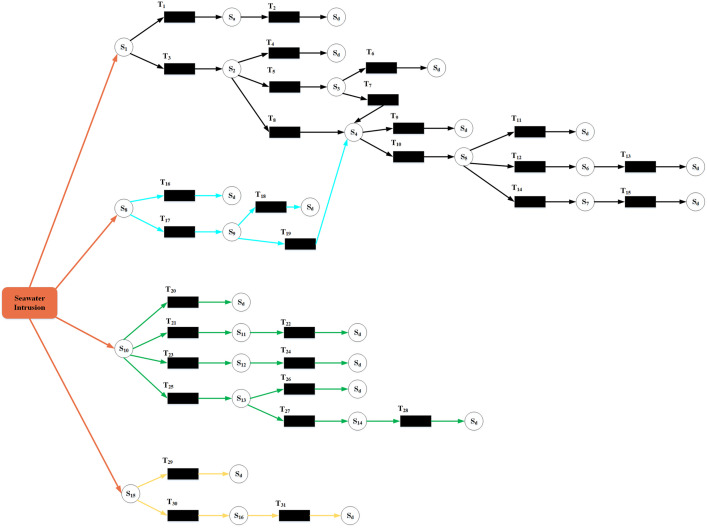
Petri net-based evolutionary path for seawater intrusion accidents in ports.

Table 2 and Fig 4 represented the accident scenario evolution paths founded on the Petri net. It should be noted that the place identifiers (S-series) labeled in Fig 4 were identical to the definition of each scenario S in Table 1. Meanwhile, the definition of each transition was constructed according to the association characteristics between scenarios. For instance, transition T_3_ corresponded to the state transfer from place S1 to place S2: S1 reflected the initial state of “Seawater erosion of concrete structure of wharf”, S2 indicated the unfavorable evolution result of “Uneven foundation settlement”, and T_3_ meant the failure of emergency measures to counteract the erosion of the concrete structure.

**Table 2:** Meaning of each transition in the Petri net.

Number	Meaning	Number	Meaning	Number	Meaning
T_1_	Emergency response effectively resists erosion of pier concrete structures	T_2_	Concrete structure restores to its original performance after re-enforcement	T_3_	Emergency response doesn’t resist erosion of pier concrete structures
T_4_	The problem of uneven foundation settlement is effectively repaired	T_5_	Uneven foundation settlement problems not repaired and large seawater retention	T_6_	Standing water is effectively removed
T_7_	Ineffective removal of standing water and more severe erosion of foundations	T_8_	Severe uneven settlement of foundations leading to structural damage to foundations	T_9_	The dock infrastructure is effectively repaired
T_10_	Further damage to the pier structure due to lack of effective repairs	T_11_	Protective devices effectively guarantee the stability of large-scale equipment	T_12_	Large-scale equipment capsizes to the sea
T_13_	Emergency response properly handles casualties on board ships at sea	T_14_	Large-scale equipment titles towards the land surface	T_15_	Emergency response adequately addresses casualties and cargo damage on the land surface
T_16_	Spare fenders are replaced in a timely manner	T_17_	Fenders aren’t replaced in a timely manner and ships remain at the pier	T_18_	No damage to the structure of the pier due to the law impact of the ship hitting the pier
T_19_	Damage to the structure due to the high impact of the ship hitting the pier	T_20_	Normal power supply is quickly restored to the power system	T_21_	Emergency power strategy for large-scale equipment fails to work
T_22_	Power supply to large-scale equipment is restored	T_23_	Emergency power strategy for shore power systems fails to work	T_24_	Power supply to shore power systems is restored
T_25_	Emergency power strategy for communication systems fails to work	T_26_	Power supply to communication systems is restored	T_27_	Structural damage to the communications system due to seawater intrusion
T_28_	Communications system is effectively repaired	T_29_	High-pole light is dealt with in a timely manner after collapsing	T_30_	Failure to deal with the collapse of the high-pole light and its landward movement occurs
T_31_	The issue of casualties and cargo damage is dealt with appropriately				

### 3.2 Application of cloud modeling

The ambiguity as well as the flexibility of qualitative language would be lost if the expert scoring method was directly applied in the assessment stage of the probability of occurrence of each transition. Therefore, the cloud model was utilized for assessment. The specific program was as follows: first, a team of experienced industry experts was formed. At the same time, a scale cloud was constructed, which served as a threshold for measuring the activation of transition. Second, founded on the scale cloud and the evaluation results of the expert team, adopted the reverse cloud generation technology, the qualitative cognition of the experts was transformed into numerical characteristic parameters to generate the transition’s judgment cloud, which served as the basis for the value range.

**Fig 5:**
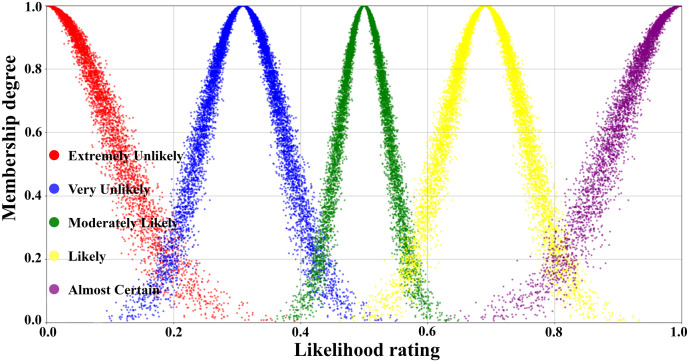
Visual presentation of cloud diagram in scale cloud.

Table 3 illustrated the parameters of the scale cloud intervals constructed based on the “3*En*” rule (Expectation *Ex*, Entropy *En*, Hyper-entropy *He*) of the cloud model. A five-level risk quantification system was utilized to express the likelihood level of transition occurrence, i.e., Extremely Unlikely, Very Unlikely, Moderately Likely, Likely, and Almost certain. In Table 3, the numerical characteristics and interval values corresponding to each risk level had been listed in detail. According to this data, combined with the forward cloud generator, a scaled cloud map containing 10000 could drop and covering the numerical domain [0,1] had been generated as displayed in Fig 5. Where the horizontal axis indicated the likelihood level of occurrence and the vertical axis reflected the affiliation of the qualitative concepts on the number field [0,1].

**Table 3:** Linguistic evaluation of the likelihood concerning risk occurrence and its scalar cloud modeling.

Chance of occurrence	Scalar cloud	
	Digital characteristic	Interval value
Extremely Unlikely	(0.000, 0.104, 0.013)	[0, 0.312]
Very Unlikely	(0.309, 0.064, 0.008)	[0.117,0.501]
Moderately Likely	(0.500, 0.039, 0.005)	[0.383,0.617]
Likely	(0.691, 0.064, 0.008)	[0.499,0.883]
Almost Certain	(1.000, 0.104, 0.013)	[0.688,1.000]

The construction of the basic data in this study primarily served to establish judgment cloud models for each transition. More specifically, 20 industry experts (including 5 university researchers, 5 frontline porty operators, 5 port enterprise managers, and 5 representatives from government regulatory authorities) were invited to assess the upper and lower bounds of the occurrence probability of each transition, thereby providing support for the development of the transition’s judging cloud models. The specific process was as follows:

(1) Based on the constructed Petri net model, the definition of each transition in the questionnaire was clarified, and the upper and lower bounds of the occurrence probability were defined as the interval [0,1].
(2) Each expert, drawing on their extensive professional experience, independently determined the occurrence threshold of each transition, generating the initial evaluation data.
(3) The collected data were subjected to validity checks: on one hand, invalid samples with uncompleted transition probability entries were excluded; on the other hand, all probability values were verified to lie within the interval [0,1], with abnormal data eliminated.
(4) Reverse cloud technology was applied to the verified valid data for calculation, so as to derive the digital features (*Ex*, *En*, *He*) of the occurrence probability of each transition.
(5) The digital features were matched and verified against the judgment cloud models, and the verification results were presented in a visualized form, enabling both qualitative description and quantitative analysis of the occurrence possibility levels of each transition.

### 3.3 Operations of Monte Carlo simulation

**Fig 6:**
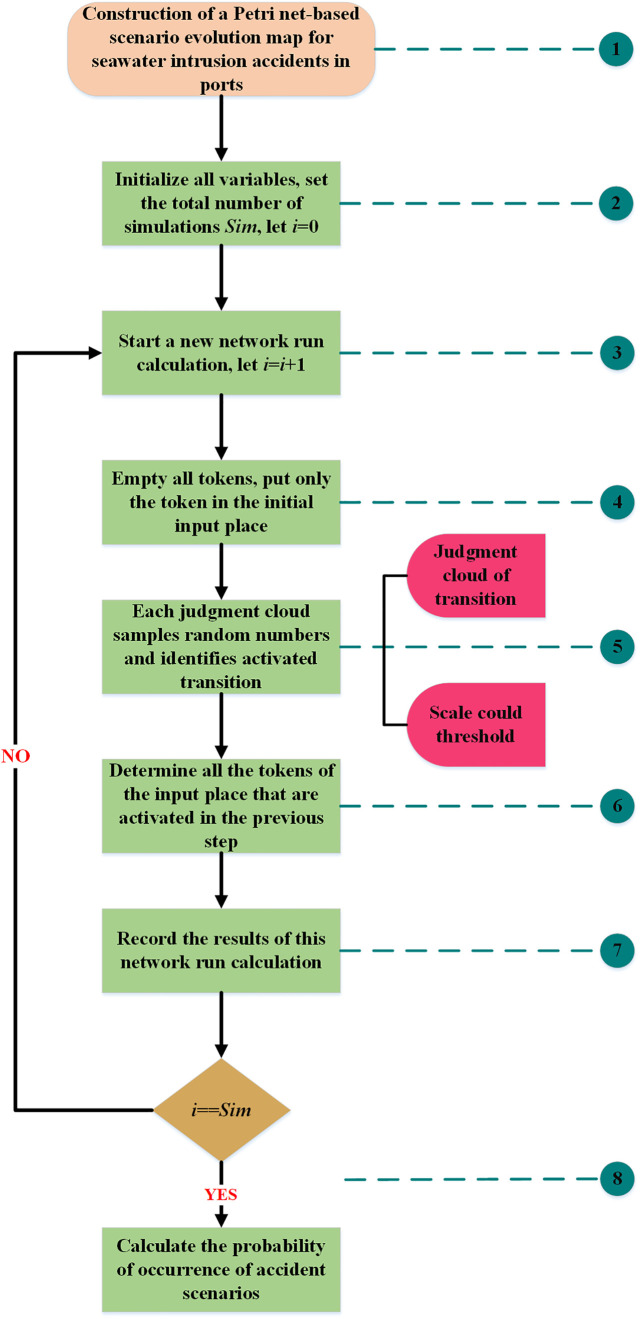
Process for quantitative assessment of seawater intrusion accident scenarios.

After completing the Petri net modeling as well as the transition probability analysis, large-scale iterative operations were performed using Monte Carlo simulation. The primary reason for choosing this method was that it enabled the reduction of contingency through multiple iterations, thereby increasing the confidence in the evaluation results. Fig 6 illustrated in the form of a flowchart how the Petri net, cloud model, and Monte Carlo simulation were applied to achieve a quantitative assessment of the evolutionary process of the scenario with regard to a seawater intrusion accident in a port.

Step 1: Transformed the scenario evolution map of port seawater intrusion accidents constructed based on the scenario element method into a Petri net scenario evolution model adapted to the needs of quantitative analysis.

Step 2: Carried out initialization settings for all variables. The total number of Monte Carlo simulations was set to 10000(Sim), and the number of operations counter *i* was initialized to 0. Meanwhile, the variable *P*_*i*_ was defined to characterize the frequency of occurrence concerning the scenario state represented by the output place after each transition activation.

Step 3: Started the Petri net calculation program. The computation was carried out sequentially from left to right according to the network topology. When a complete round of scenario evolution simulation was executed, the value of the counter *i* was increased by 1 accordingly.

Step 4: Emptied the tokens retained by the place at the end of the previous round of computation. Founded on the updated parameter configurations, initiated a new round of scenario evolutionary push.

Step 5: Determined the transition activation status by conditional function F. First, for each transition, a sample of values was randomly generated from its corresponding judgment cloud. Then, the value was compared with the scale cloud likelihood class interval that was calibrated by expert experience. If the sample value was exactly within the preset quantization interval, the transition was determined to satisfy the activation condition. The expression for the condition function F was:


$$F=\left\{ \;\;\;\begin{array}{c} true,Bi\left(\min\right)\leq normarnd\left(Ci\right)\leq Bi\left(\max\right)\\ falese,normarnd\left(Ci\right)<Bi\left(\min\right)orBi\left(\max\right)<normarnd\left(Ci\right) \end{array}\right.$$ (1)

where, *C*_*i*_ expresses the judgment cloud of the transition; *normarnd*(*C*_*i*_) indicates a random value selected from the judgment cloud; *B*_*i*_() represents the interval value corresponding to the risk likelihood level, *B*_*i*_(*min*) and *B*_*i*_(*max*) are the interval minimum and maximum values, respectively.

Step 6: After confirming the activation of the transition, it was necessary to check whether its corresponding output place had a token. The purpose of this operation was to clarify whether the scenario state represented by the place had been reached or not, to ensure the coherence of the scenario evolution process. During this process, the number of the token in the input place remained unchanged, while the number of tokens in the output place was increased by one accordingly.

Step 7: After completing a round of Petri net operations, the current results were recorded.

Step 8: Compared the number of times i had been executed with the present total number of simulations Sim: if the specified total number of simulations wasn’t reached, started the simulation process again and returned to step 3; of the specified number of times had been reached, terminated the cyclic operation and count the probability of the occurrence of the scenario state in the evolution process.

## 4 Quantitative calculation results and analysis

### 4.1 Transition judgment cloud and its scala cloud threshold

**Fig 7:**
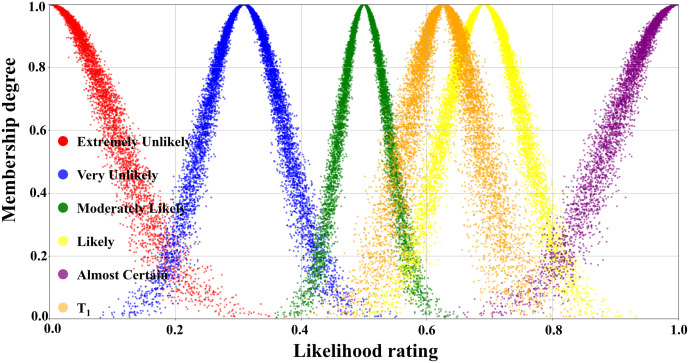
Judgmental cloud diagrams for transition T_1._

**Fig 8:**
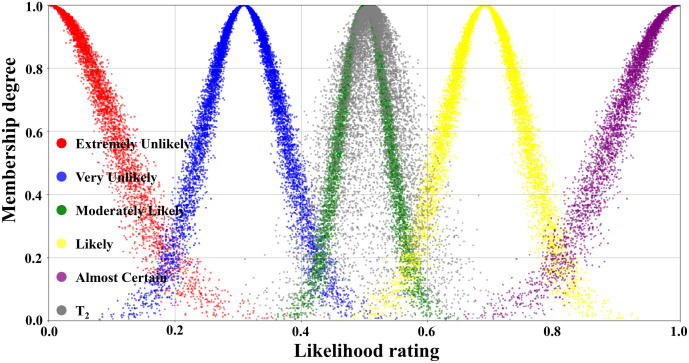
Judgmental cloud diagrams for transition T_2._

**Fig 9:**
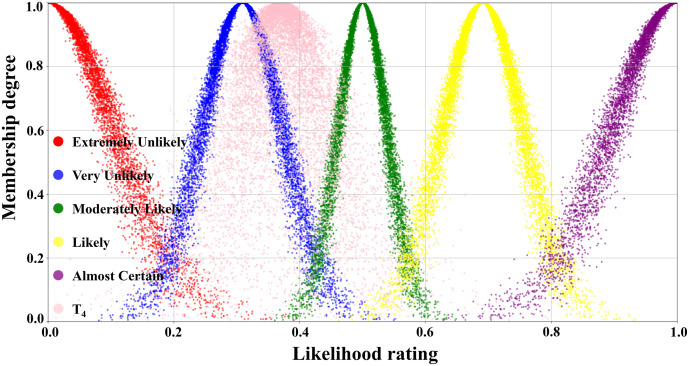
Judgmental cloud diagrams for transition T_4._

**Fig 10:**
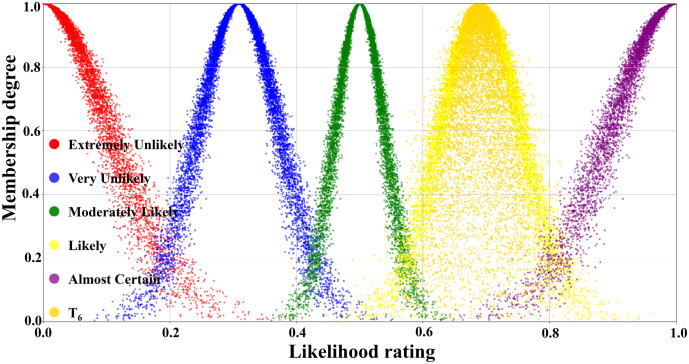
Judgmental cloud diagrams for transition T_6._

According to the assessment results of the expert group, the numerical characteristics of each transition were obtained by the inverse cloud generator, as displayed in Table 4. Because a total of 31 transitions were involved in the evolution process, limited by the length of the paper, the transitions with certain representativeness, namely, T_1_, T_2_, T_4_, and T_6_, were selected for the visual display of the judgmental cloud diagrams, which were shown in detail in Fig 7, Figure 8, Fig 9, and Fig 10.

**Table 4:** Numerical characterization of transition judgment cloud model.

Transition	Judgment cloud model C_*i*_	Transition	Judgment cloud model C_*i*_
T_1_	C_1_(0.626, 0.056, 0.014)	T_2_	C_2_(0.509, 0.04, 0.023)
T_3_	C_3_(0.519, 0.096, 0.014)	T_4_	C_4_(0.37, 0.061, 0.041)
T_5_	C_5_(0.51, 0.12, 0.04)	T_6_	C_6_(0.689, 0.035, 0.025)
T_7_	C_7_(0.236, 0.097, 0.012)	T_8_	C_8_(0.231, 0.077, 0.021)
T_9_	C_9_(0.599, 0.093, 0.039)	T_10_	C_10_(0.478, 0.076, 0.029)
T_11_	C_11_(0.420 0.041 0.009)	T_12_	C_12_(0.554, 0.11, 0.024)
T_13_	C_13_(0.537, 0.09, 0.023)	T_14_	C_14_(0.619, 0.093, 0.039)
T_15_	C_15_(0.421, 0.044, 0.008)	T_16_	C_16_(0.713, 0.071, 0.041)
T_17_	C_17_(0.679, 0.093, 0.039)	T_18_	C_18_(0.721, 0.077, 0.023)
T_19_	C_19_(0.504, 0.052, 0.009)	T_20_	C_20_(0.292, 0.078, 0.024)
T_21_	C_21_(0.804, 0.079, 0.011)	T_22_	C_22_(0.613, 0.058, 0.021)
T_23_	C_23_(0.677, 0.041, 0.01)	T_24_	C_24_(0.34, 0.064, 0.009)
T_25_	C_25_(0.504, 0.054, 0.009)	T_26_	C_26_(0.409, 0.085, 0.031)
T_27_	C_27_(0.747, 0.051, 0.007)	T_28_	C_28_(0.27, 0.064, 0.009)
T_29_	C_29_(0.649 0.058 0.018)	T_30_	C_30_(0.482, 0.05, 0.016)
T_31_	C_31_(0.648, 0.059, 0.019)		

Fig 7 illustrated the judgment cloud diagram of the transition T_1_. It could be seen that the 10000 generated cloud droplets were mainly clustered at the junction of the “Moderately Likely” and “Likely” intervals, but the overall distribution was obviously skewed towards the “Likely” side. Founded on the distribution characteristics of the cloud diagram, the expert group finally concluded that the occurrence level of transition T_1_ (Emergency response effectively resists erosion of pier concrete structures) was rated as “Likely”.

In contrast, the T_2_ judgment cloud in Fig 8 represented that the same number of 10000 droplets were primarily concentrated in the “Moderately Likely” interval and highly coincided with it. This indicated that the expert group reached a high degree of consensus on the probability of T_2_’s occurrence (Concrete structure restores to its original performance after re-enforcement), which was rated as “Moderately Likely”.

The judgmental cloud diagram for transition T_4_ was expressed in Fig 9. The cloud drops were mainly clustered in the “Very Likely” range, but the data were more dispersed. This demonstrated that although the final assessment result showed that the likelihood level of transition T_4_ was “Very Unlikely”, there was a great deal of disagreement within the expert group. On the contrary, in the judgment cloud diagram of transition T_6_, the cloud drops represented a higher degree of aggregation, as displayed in Fig 10. This reflected that the overall opinion of the expert group had been unified, and the general opinion was that the occurrence level of transition T_6_ was at the “Likely” level. Through the combination of the scale cloud and the judgment cloud, the cloud model not only realized the intuitive visualization of the likelihood of the occurrence of each transition, but also clearly reflected the discrete characteristics of the expert group’s evaluation results.

Table 5 detailed the likelihood classes of the 31 transition and their corresponding interval boundary values. The data in Table 4 and Table 5 were, in fact, the specific embodiment of the transition judgment cloud and the threshold values of the scale cloud in the flowchart shown in Fig 6. In the quantitative calculation process concerning scenario evolution, by analyzing the response relationship between the sets of data (i.e., whether the random numbers generated by the judgment cloud fell into the threshold intervals of the scale cloud), the activation state of the corresponding transition was determined. Then, the evolution path of the relevant accident scenarios and their probability of occurrence were deduced.

**Table 5:** T_1_-T_31_ transition in scale-cloud based threshold selection.

Transition	Rank	Interval value	Transition	Rank	Interval value
T_1_	Likely	[0.499, 0.883]	T_2_	Moderately Likely	[0.383, 0.617]
T_3_	Moderately Likely	[0.383, 0.617]	T_4_	Very Unlikely	[0.117, 0.501]
T_5_	Moderately Likely	[0.383, 0.617]	T_6_	Likely	[0.499, 0.883]
T_7_	Very Unlikely	[0.117, 0.501]	T_8_	Very Unlikely	[0.117, 0.501]
T_9_	Moderately Likely	[0.383, 0.617]	T_10_	Moderately Likely	[0.383, 0.617]
T_11_	Moderately Likely	[0.383, 0.617]	T_12_	Moderately Likely	[0.383, 0.617]
T_13_	Moderately Likely	[0.383, 0.617]	T_14_	Likely	[0.499, 0.883]
T_15_	Moderately Likely	[0.383, 0.617]	T_16_	Likely	[0.499, 0.883]
T_17_	Likely	[0.499, 0.883]	T_18_	Likely	[0.499, 0.883]
T_19_	Very Unlikely	[0.117, 0.501]	T_20_	Moderately Likely	[0.383, 0.617]
T_21_	Likely	[0.499, 0.883]	T_22_	Likely	[0.499, 0.883]
T_23_	Likely	[0.499, 0.883]	T_24_	Very Unlikely	[0.117, 0.501]
T_25_	Moderately Likely	[0.383, 0.617]	T_26_	Moderately Likely	[0.383, 0.617]
T_27_	Likely	[0.499, 0.883]	T_28_	Very Unlikely	[0.117, 0.501]
T_29_	Likely	[0.499, 0.883]	T_30_	Moderately Likely	[0.383, 0.617]
T_31_	Likely	[0.499, 0.883]			

### 4.2 Risk probability and analysis of seawater intrusion accident scenarios

Founded on the data provided in Section 4.1, Petri net, cloud model, and Monte Carlo simulation were applied to quantitatively calculate the scenario evolution of a seawater intrusion accident in ports. To ensure the stability of the stability of the scenario risk probability, the average of the three independent simulation results was selected as the final basis for the probability. This treatment ensured the reliability of the final results while taking into account their repeatability. The risk occurrence probabilities of distinct accident scenarios were expressed in Table 6.

**Table 6:** Probability of occurrence for scenario evolution concerning seawater intrusion accidents in ports.

Scenario	Probability of occurrence	Scenario	Probability of occurrence
S2	20.3%	S3	3.3%
S4	5.9%	S5	1.2%
S6	0.4%	S7	0.4%
S9	14.2%	S11	57.2%
S12	50.4%	S13	36.1%
S14	22.4%	S16	53.9%

In the data presented in Table 6, there was a significant difference in the probability of occurrence concerning distinct accident scenarios (S1 File). Among them, the highest probability reached 57.2%, and the lowest was only 0.4%, which was a momentous polarization. For the sake of analyzing the probability of occurrence with regard to accident scenarios more deeply, they were divided into three tiers for discussion.

**Fig 11:**
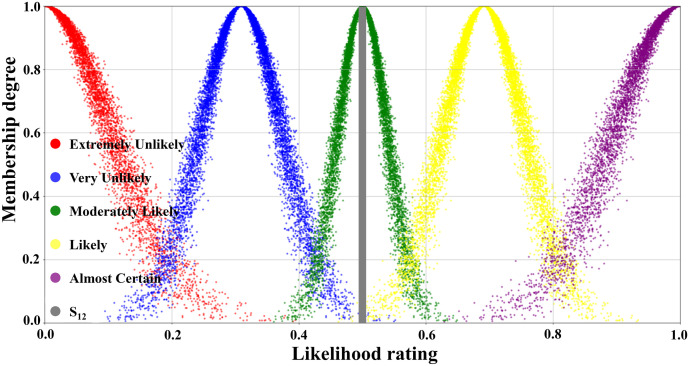
Visualization of scenario S12 in the scale cloud.

First, the first tier was in the 40%−60% range, covering Scenarios S11, S16, and S12, with corresponding probability values of 57.2%, 53.9%, and 50.4%, respectively, which were in the top three of the occurrence probabilities. According to the qualitative analysis of the scale cloud framework, these three scenarios were categorized as “Likely” to occur. In favor of enhancing the intuition of the analysis, scenario S12 (Failure of shore power system affecting normal operation) was selected as an example for visualization, as represented in Fig 11.

**Fig 12:**
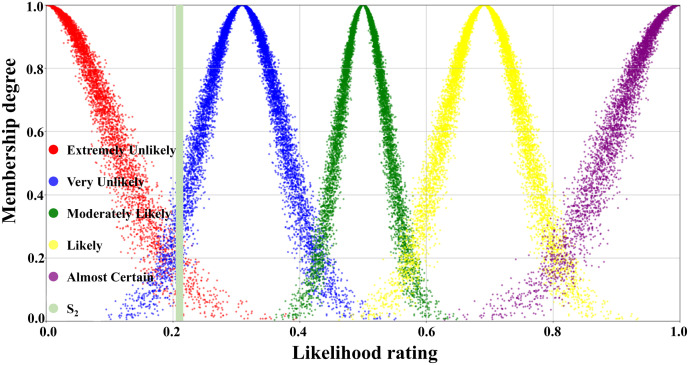
Visualization of scenario S2 in the scale cloud.

The second tier corresponded to the probability range of 20%−40%, including scenarios S13, S14, and S2. The quantitatively calculated probabilities of occurrence for the three scenarios were 36.1%, 22.4%, and 20.3%, respectively. Fig 12 illustrated the location of scenario S2 (Uneven foundation settlement) in the scale cloud, which was slightly above 20% and fell in the overall “Very Unlikely” occurrence class. It was worth noting that although the probability value of scenario S13 was close to the upper limit of the range, all three scenarios were categorized as “Very Unlikely” based on the scale cloud criteria.

**Fig 13:**
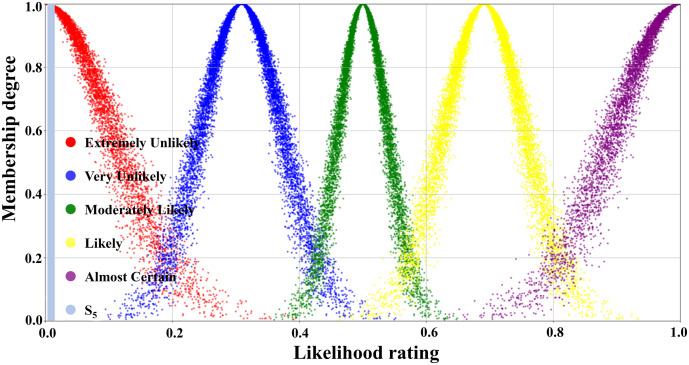
Visualization of scenario S5 in the scale cloud.

Tier 3 corresponded to a probability interval of 0–20% and encompassed a total of six accident scenarios (S3, S4, S5, S6, S7, S9). There were notable differences in the probability of occurrence concerning each scenario within this tier, with the distribution spanning from 0.4% to 14.2%. As shown in Fig 13, scenario S5 was categorized as “Extremely Unlikely” with a probability of occurrence of 1.2%. In the same tier, scenarios S6 (Large-scale loading and unloading equipment tipping over to the sea surface) and S7 (Large-scale loading and unloading equipment tipping over to the land surface) expressed a very low probability of occurrence of 0.4%.

## 5 Conclusion

In this paper, the evolution process of seawater intrusion accident scenarios in ports was systematically deduced by applying the scenario elements method, and the quantitative judgment of evolution paths was realized through Petri net, cloud model, and Monte Carlo simulation, and the distribution characteristics of the probability concerning distinct scenarios’ occurrence were clarified. The main research results were as follows:

(1) Founded on the disaster system framework composed of disaster-causing body, disaster-affected body, and disaster-resistant body, the scenario evolution model after intrusion accident in ports was constructed utilizing scenario elements analysis. Typical accident scenarios such as concrete structure erosion, uneven foundation settlement, fender displacement or fracture, and power supply interruption were identified. Meanwhile, the triggering relationship and dynamic evolution law between distinct accident scenarios were revealed.
(2) The Petri net theory was applied to transform the existing scenario evolution, and a Petri net model containing 31 transitions was constructed. By combining the inverse cloud and forward cloud, for distinct transitions, the judgment cloud model and the scale cloud threshold were constructed, realizing the transformation of qualitative cognition and quantitative expression. On this basis, the Monte Carlo simulation was utilized to realize the iterative operation of ten thousand times to ensure the reliability of the output results.
(3) According to the quantitative results, the probability grading system of the accident scenario was formed: the probability of occurrence concerning S11(57.2%), S12(50.4%), and S16(53.9%) were in the range of 40%−60%, which belonged to the grade of “Moderately Likely”; the probability of occurrence with regard to S13, S14, and S2 was in the range of 20%−40%, which was categorized as “Very Unlikely”; the remaining scenarios were uniformly classified into the grade of “Extremely Unlikely”, with probabilities that didn’t exceed the 20% threshold. Among them, the probabilities of S6 and S7 were only 0.4%, ranking the bottom of the grading system.

## Supporting information

S1 File Raw data. (DOCX)
