# Supplementary material for: Scenario evolution modeling and probabilistic assessment of seawater intrusion accident in ports: An integrated framework combining disaster theory and multi-method simulation
Source: PLoS One. 2025 Oct 31;20(10):e0334696. doi: 10.1371/journal.pone.0334696 (PMC12578212; doi:10.1371/journal.pone.0334696)
Supplement: S1 File — (DOCX) [file pone.0334696.s001.docx]

**Assessment of upper and lower limits of occurrence probability for each transition in the scenario evolution of port seawater intrusion accidents**

**Name:**

**Occupation:**

**Work Unit:**

**Probability range: [0,1]**

| Transition | Definition | Upper limit of occurrence probability | Lower limit of occurrence probability |
| --- | --- | --- | --- |
| T_1_ | Emergency response effectively resists erosion of pier concrete structures |  |  |
| T_2_ | Concrete structure restores to its original performance after re-enforcement |  |  |
| T_3_ | Emergency response doesn’t resist erosion of pier concrete structures |  |  |
| T_4_ | The problem of uneven foundation settlement is effectively repaired |  |  |
| T_5_ | Uneven foundation settlement problems not repaired and large seawater retention |  |  |
| T_6_ | Standing water is effectively removed |  |  |
| T_7_ | Ineffective removal of standing water and more severe erosion of foundations |  |  |
| T_8_ | Severe uneven settlement of foundations leading to structural damage to foundations |  |  |
| T_9_ | The dock infrastructure is effectively repaired |  |  |
| T_10_ | Further damage to the pier structure due to lack of effective repairs |  |  |
| T_11_ | Protective devices effectively guarantee the stability of large-scale equipment |  |  |
| T_12_ | Large-scale equipment capsizes to the sea |  |  |
| T_13_ | Emergency response properly handles casualties on board ships at sea |  |  |
| T_14_ | Large-scale equipment titles towards the land surface |  |  |
| T_15_ | Emergency response adequately addresses casualties and cargo damage on the land surface |  |  |
| T_16_ | Spare fenders are replaced in a timely manner |  |  |
| T_17_ | Fenders aren’t replaced in a timely manner and ships remain at the pier |  |  |
| T_18_ | No damage to the structure of the pier due to the law impact of the ship hitting the pier |  |  |
| T_19_ | Damage to the structure due to the high impact of the ship hitting the pier |  |  |
| T_20_ | Normal power supply is quickly restored to the power system |  |  |
| T_21_ | Emergency power strategy for large-scale equipment fails to work |  |  |
| T_22_ | Power supply to large-scale equipment is restored |  |  |
| T_23_ | Emergency power strategy for shore power systems fails to work |  |  |
| T_24_ | Power supply to shore power systems is restored |  |  |
| T_25_ | Emergency power strategy for communication systems fails to work |  |  |
| T_26_ | Power supply to communication systems is restored |  |  |
| T_27_ | Structural damage to the communications system due to seawater intrusion |  |  |
| T_28_ | Communications system is effectively repaired |  |  |
| T_29_ | High-pole light is dealt with in a timely manner after collapsing |  |  |
| T_30_ | Failure to deal with the collapse of the high-pole light and its landward movement occurs |  |  |
| T_31_ | The issue of casualties and cargo damage is dealt with appropriately |  |  |

**Note: The survey data will be used for scientific research. If you agree, please sign to confirm at the end of the questionnaire.**

**Signature:**
